# The effect of mother’s age and other related factors on neonatal survival associated with first and second birth in rural, Tanzania: evidence from Ifakara health and demographic surveillance system in rural Tanzania

**DOI:** 10.1186/1471-2393-14-240

**Published:** 2014-07-22

**Authors:** Majige Selemani, Mathew A Mwanyangala, Sigilbert Mrema, Amri Shamte, Dan Kajungu, Abdallah Mkopi, Michael Johnson Mahande, Rose Nathan

**Affiliations:** 1 Ifakara Health Institute, (IHI), Plot 463, Kiko Avenue, off Old Bagamoyo Road, Mikocheni P.O Box 78373, Dar es Salaam, Tanzania; 2 INDEPTH Network, P.O Box KD 213, Kanda, Accra, Ghana; 3 Santé Stat Analytical Research Institute (SSARI), P.O Box 37193, Kampala, Uganda; 4 Department of Statistics, University of Dar es Salaam, P.O. Box 35091, Dar es Salaam, Tanzania; 5 Department of Epidemiology & Applied Biostatistics, Kilimanjaro Christian Medical University College, Moshi, Tanzania

**Keywords:** Neonates, Neonatal mortality, Teenagers, Birth order, Birth interval

## Abstract

**Background:**

With a view to improve neonatal survival, data on birth outcomes are critical for planning maternal and child health care services. We present information on neonatal survival from Ifakara Health and Demographic Surveillance System (HDSS) in Tanzania, regarding the influence of mother’s age and other related factors on neonatal survival of first and second births.

**Methods:**

The study conducted analysis using longitudinal health and demographic data collected from Ifakara HDSS in parts of Kilombero and Ulanga districts in Morogoro region. The analysis included first and second live births that occurred within six years (2004–2009) and the unit of observation was a live birth. A logistic regression model was used to assess the influence of socio-demographic factors on neonates’ survival.

**Results:**

A total of 18,139 first and second live births were analyzed. We found neonatal mortality rate of 32 per 1000 live births (95% CI: 29/1000-34/1000). Results from logistic regression model indicated increase in risk of neonatal mortality among neonates those born to young mothers aged 13–19 years compared with those whose mother‘s aged 20–34 years (aOR = 1.64, 95% CI = 1.34-2.02). We also found that neonates in second birth order were more likely to die than those in first birth order (aOR = 1.85: 95% CI = 1.52-2.26). The risk of neonatal mortality among offspring of women who had a partner co-resident was 18% times lower as compared with offspring of mothers without a partner co-resident in the household (aOR = 0.82: 95% CI = 0.66-0.98). Short birth interval (<33 months) was associated with increased risk of neonatal mortality (aOR = 1.50, 95% CI =1.16-1.96) compared with long birth interval (> = 33 months). Male born neonates were found to have an increased risk (aOR = 1.34, 95% CI =1.13- 1.58) of neonatal mortality as compared to their female counterparts.

**Conclusions:**

Delaying the age at first birth may be a valuable strategy to promote and improve neonatal health and survival. Moreover, birth order, birth interval, mother’s partner co-residence and sex of the neonate appeared as important markers for neonatal survival.

## Background

It is estimated that about 2.96 million infants worldwide die each year during first four weeks of life (neonatal period) [1]. About 98% of these deaths occur in low- and middle-income countries; with the highest proportion in sub-Saharan Africa and south-east Asia [1]. In 2012, neonatal mortality accounted for 43% of the under five deaths in developing countries [1,2]. A recent Tanzania Demographic and Health Survey report showed a decline in under-five mortality rate from 112 per 1000 live births in 2004/05 to 81 per 1000 live births in 2010 [3]. Similarly, the infant mortality rate has dropped from 68 to 45 per 1000 live births during the same period. However, the share of neonatal death has slowly dropped from 32 to 25 per 1000 live births during the survey period 2004/05 to 2010. Reduction in neonatal deaths is an essential step towards effort to accelerate progress towards achievement of MDG 4 for child survival. However, this requires better understanding of major causes and risk factors for neonatal deaths.

Several risk factors have been suggested to influence neonatal mortality including maternal age, parity, race, smoking birth weight, gestational age, labour complications, number of antenatal care visits, previous unfavourable outcomes (stillbirth and neonatal death) and various socio-economic factors [4,5].

Pregnancy during teenage is a significant problem globally, with the highest incidence rates occurring in developing nations [6]. Tanzania is among the countries with highest teenage pregnancy (44%) and birth rates in the world [7]. National survey reports indicate that about 25% of girls begun child-bearing at 17 years; this figure increases to almost 40 per cent by age 18 [3,7]. While early childbearing has often been regarded as a social issue, there is evidence that young maternal age may be linked to adverse pregnancy outcomes including low birth weight (LBW), preterm birth, intrauterine growth restriction, stillbirth, neonatal mortality [5]. Other studies have shown relationship between birth orders, birth interval, maternal age and neonatal mortality [8-10]. Previous studies in Tanzania that concentrated on effect of maternal age on neonatal outcome had small sample sizes which are difficult to make inference. Yet, there is scant information about the influence of mothers’ age, birth order, and birth interval on neonatal mortality for the first and second births in Tanzania.

In view of the importance of neonatal survival, epidemiological data regarding the influence of mother’s age on neonatal mortality provide important information to policy makers and program managers when designing interventions to reduce neonatal mortality [1,7]. Therefore, this paper used longitudinal data generated in Health and Demographic Surveillance Systems to study the influence of mother’s age and other factors on neonatal survival of first and second births in the rural part of south-eastern Tanzania.

## Methods

### Study design and setting

The analysis used data from Ifakara Health and Demographic Surveillance Site (HDSS) situated in Kilombero and Ulanga districts in Morogoro region. In 2011, the total population under surveillance was over 100,000 living in 16,000 households [11]. The Ifakara HDSS has consistently been recording pregnancies, pregnancy outcomes, deaths and migrations by visiting households once every four months since 1997 after completion of the baseline census carried out in 1996. Since then every household in the surveillance area has been visited by a trained interviewer every 4 months to record pregnancies, pregnancy outcomes, deaths and migrations that have happened since the previous visit. Household registers are used to record each of those events. All registered deaths are followed up with a verbal Autopsy (VA) by well trained field staff. Date of birth of each individual is included in the household registers and each event is recorded along with specific date it happened. Place of delivery and place of death are recorded as health facility, home or elsewhere.

The population is predominantly rural and ethnically heterogeneous. Majority of the households earn their living from subsistence farming, few are engaged in fishing and small-scale trading. The population of the study area is served by a network of health facilities, at the time of the study there were two hospitals, four health centres and twenty one dispensaries in Kilombero district; two hospitals, three health centres and twenty dispensaries in Ulanga district.

Overall, the total fertility rate in the study area is four children per women in their lifetime. Within the study population, about 60% of all deliveries occur in health facilities mainly in dispensaries. In the study area, most (96%) of pregnant women attend at least one antenatal care (ANC) visit from the skilled birth attendants. However, only 43% of pregnant women are recorded with at least four visits. At the time of study, continuum of care was not fully introduced in the study area.

Data credibility was ensured at all stages of collection and processing to enhance quality. Up to 5% of randomly selected households were visited by field supervisors for repeated interviews. Other strategies included accompanied interviews as well as surprise field visits by field supervisor. Data management is done using the household-registration system (HRS 2) with built in consistency and range checks. Detailed description of the study area is presented elsewhere [12].

### Data processing and analysis

This paper reports analysis using longitudinal data collected in the Ifakara Health and Demographic Surveillance Site (IHDSS) for children born between 2004 and 2009. First and second birth order of neonates were extracted from the database including their survival status within the first 28 days of life. All women who had first and second live births that occurred between 2004 and 2009 were included in the analysis. We used live births as the unit of observation. Explanatory variables include: age of mother, sex, birth order, birth interval, place of delivery, social economic status (SES), education of the mother, mother having partner co-resident, and season of the year. The outcome variable was neonatal death.

Birth order was classified into two groups - first birth order and second birth order. Birth interval among second birth was classified as long birth interval (greater than 33 months after the previous birth) and short birth interval (less than 33 months after the previous birth), as per WHO recommendation [13]. The age of mother at delivery was categorized into three groups teenage (less than 20 years), (20 to 34 years) and (35 to 49 years) [14]. The place of delivery was classified into two groups: health facility and outside health facility. All deliveries that occurred on the way to a health facility, home or elsewhere outside health facilities were all classified as outside health facility deliveries. Seasons of delivery were classified into two groups: dry (June-October) and wet (November-May) according to the dates of birth that correspond to the seasons of the year in the study area. Other variables were maternal educational level which was classified into two groups, “primary level or below” and “above primary education”, sex of neonate and mother’s partner co-resident.

Household wealth status was constructed using Principal Component Analysis (PCA) [15]. Items included in the PCA are household assets; animals, TV, bicycle and radio. Also type of toilet, source of drinking water, house roofing material, wall material, and floor material were included in PCA. Finally, all households were grouped into five categories: Poorest, Poorer, Poor, Less Poor or Least Poor according to their household wealth score. The outcome variable was defined by assigning the neonatal death into one of the two categories: “1” if a newborn die within the first 28 days of life and “0” if a newborn had not die within the first 28 days of the life.

### Statistical methods

The analysis used both descriptive and analytical statistics. Neonatal death rates by each variable were calculated and presented. Pearson’s Chi-Square test was used to determine the association between a set of explanatory variables and neonatal death for categorical variables. Further analysis was performed in multivariable logistic regression model to assess relative effect of the variables hypothesized to influence neonatal death. The Cox proportional hazard regression model could not be used because the number of neonatal deaths were less than four percent, thus more than 96% would not observe the event in the pre-designated time interval and would be treated as censored (or missing).

Explanatory variables were selected for inclusion in the multivariate logistic regression if the p value was less than 0.1 p-values in the univariate analysis and improved the overall model [16]. The model was checked for statistical interactions and adequacy before being approved as final. An alpha level of 0.05 was used for all tests of statistical significance.

Statistical analyses were performed using STATA version 11.0 [17]. Odd ratios (OR) with 95% confidence intervals (CI) were used as measures of strength of association. Given the low prevalence of neonatal mortality, the odds ratios are close approximation of relative risks. The neonatal mortality rate was calculated as the number of neonatal deaths divided by number of live births in a given year and expressed per 1000 live births.

### Ethical approval

Ifakara Health and Demographic Surveillance System were established with an initial aim of evaluating the effect of a large-scale social marketing of insecticide-treated nets on child survival in rural Tanzania. The Ethical clearance was granted by the Medical Research Coordinating Committee (MRCC) of the National Institute for Medical Research (NIMR) in Tanzania. For each household visit, verbal consent was sought from the respondent.

## Results

### Demographic characteristics of live birth

The analysis was based on 16,000 households registered in the Health and Demographic Surveillance System. A total of 11, 562 that had at least one live birth during the study period were included in the analysis, there was no refusal.

A total of 18,139 first and second live births were recorded in the Ifakara Health and Demographic Surveillance Area for the period from January 2004 to December, 2009. Of these, more than half 9,172 (51%) were males. A total of 576 neonatal deaths occurred during the study period, this translates into a neonatal mortality rate of 32 per 1000 live births (95% CI: 29/1000-34/1000).

### Neonatal mortality rate

The risk of neonatal mortality rate among all live births reached its peak of 35 per 1000 live births in the year 2005, it then decreased to a lowest mortality level of 29 per 1000 live births in 2009. We observed a marked decline in neonatal mortality rates for the period from 2006 to 2009 (Table 1).

**Table 1:** Estimates of neonatal mortality for the year of 2004-2009 (N = 18,139)

	**Live birth**			**Neonatal Mortality**		
***Yea*** ***r***	**No. live births**	**Deaths**	**Percent**	**Rate per 1000 live births**	**95% CI**	**P-value**
**2004-09**	**18,139**	**576**	**3.18**	**31.8**	**29.3-34.4**	0.640
2004	2,734	87	3.08	30.8	24.5-37.2	
2005	2,831	105	3.58	35.8	29.0-42.5	
2006	2,869	102	3.43	34.3	27.8-40.9	
2007	2,877	92	3.10	31.0	24.8-37.2	
2008	3,232	99	2.97	29.7	24.0-35.5	
2009	3,020	91	2.93	29.3	23.3-35.2	

**Figure 1:**
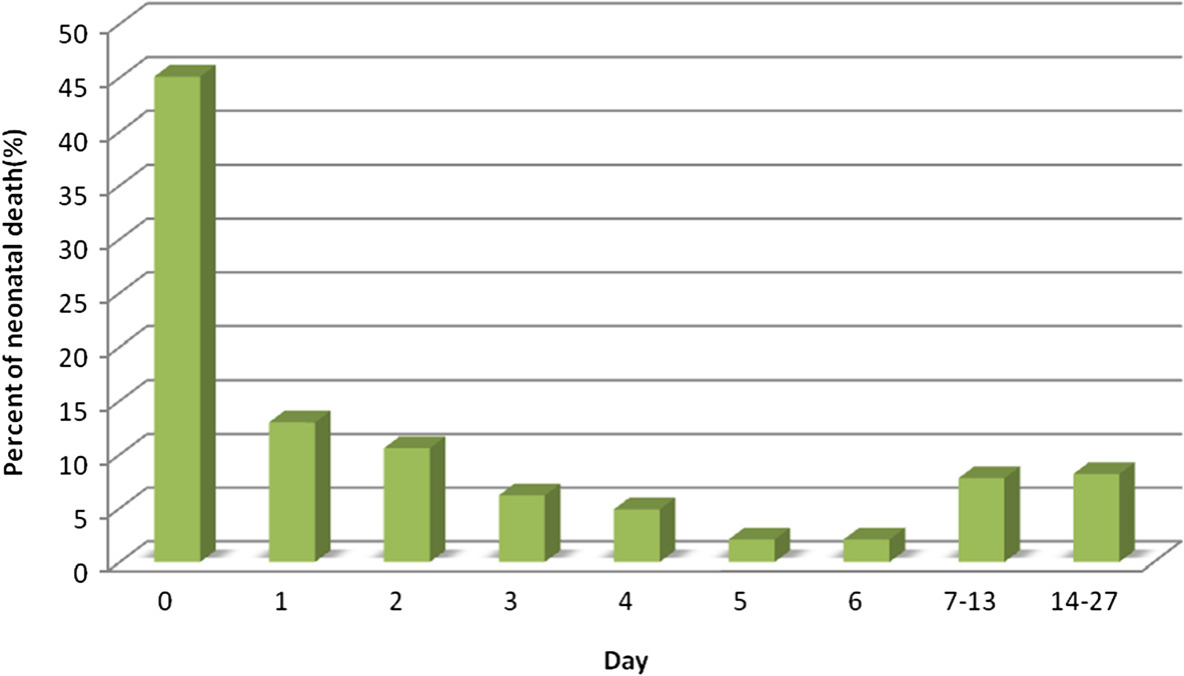
Timing of the neonatal deaths.

In this study, new-borns for teenage mothers (13–19 years) had higher neonatal mortality rate as compared with those born to older mothers (20 to 34 years) and (35 to 49 years), (46 per 1,000 live births, 28 and 31 per 1,000 live births respectively) (Table 2). In regards to birth order, we found higher neonatal mortality rates among second birth order as compared with first birth order (38 vs 28 per 1000 live births, respectively). Our results also showed that short birth intervals compared with long birth intervals were associated with an increased neonatal mortality rate in second births (47 and 29 per 1000 live birth respectively). Neonates born to mothers who had their partners co-resident had a lower mortality compared with those whose mothers had no partners (30 vs 38 per 1000 live births). Our data suggests that teenager mothers’ with a short birth interval after the previous birth have excess neonatal deaths (90 per 1000 live births) as compared with their counterparts with long birth interval (Table 3). Of the 576 live births that resulted in neonatal deaths, 45% happened on the same day of birth and 84% occurred during the first week of life (Figure 1).

**Table 2:** Distribution of neonatal deaths by explanatory variables (N = 18,139)

**Factors**	**No. live births**	**Death n/N(%)**	**Rate per 1000 live births**	**95% CI**	**P-value**
***Sex***	**18,139**	**576/18,139(3.18)**	**31.8**	**29.3-34.4**	**<0.01**
Male	9,172	331/9,172(3.61)	36.1	32.3-39.9	
Female	8,967	245/8,967(2.71)	27.3	23.9-30.7	
***Mothers’ age group***	18,139	**576/18,139(3.18)**	**31.8**	**29.3-34.4**	**<0.01**
Teenage(13–19)	3,238	150/3,238(4.63)	46.3	39.1-53.6	
Non Teenage(20–34)	12,294	346/12,294(2.81)	28.1	25.2-31.1	
Non teenage(35–49)	2,607	80/2,607(3.07)	30.7	24.1-37.3	
***Birth order***	**18,139**	**576/18,139(3.18)**	**31.8**	**29.3-34.4**	**<0.01**
First order	11,562	325/11,562(2.81)	28.1	25.1-31.1	
Second order	6,577	251/6,577(3.82)	38.2	33.5-42.8	
**Interval between first & second birth**	**6,577**	**251/6,577(3.82)**	**38.2**	**33.6-43.1**	**<0.01**
Short birth interval(less than 33 months after the previous birth)	3,341	156/3341(4.67)	46.7	39.5-53.8	
Long birth interval(greater than 33 months after the previous birth)	3,236	95/3236(2.94)	29.4	23.5-35.2	
***SES at birth***	**18,139**	**576/18,139(3.18)**	**31.8**	**29.3-34.4**	**0.762**
Poorest	3,710	121/3710(3.26)	32.6	26.8-38.3	
Poorer	3,628	110/3628(3.03)	30.3	24.7-35.9	
Poor	3,861	119/3861(3.08)	30.8	25.4-36.3	
Less Poor	3,780	117/3780(3.10)	31	25.4-36.5	
Least Poor	3,160	109/3160(3.45)	34.5	28.1-40.9	
***Place of delivery***	**18,139**	**576/18,139(3.18)**	**31.8**	**29.3-34.4**	**0.842**
Outside health facility	7,485	240/7485(3.21)	32.1	28.1-36.1	
Health Facility	10,654	336/10654(3.15)	31.5	28.2-34.9	
***Education of mothers’***	**18,139**	**576/18,139(3.18)**	**31.8**	**29.3-34.4**	**0.072**
Above primary education	3,220	86/3221(2.67)	26.7	21.4-32.9	
Below and Primary education	14,919	490/14919(3.28)	32.8	28.0-34.1	
**Mother having partner**	**14,912**	**576/18,139(3.18)**	**31.8**	**29.3-34.4**	**0.031**
Present	14,912	454/14912(3.04)	30.4	27.7-33.2	
Absent	3,227	122/3227(3.78)	37.8	31.2-44.4	
**Season**	**18,139**	**576/18,139(3.18)**	**31.8**	**29.3-34.4**	**0.813**
Dry season	7,817	251/7817(3.21)	32.1	28.3-36.3	
Wet season	10,322	325/10,322(3.15)	3.15	28.2-35.0	

P-values are based on Pearson’s Chi-Square test at 5% significance level.

**Table 3:** Distribution of neonatal deaths by mother’s age against birth order and birth interval

	**Mother's age**								
	**13-19**		**20-34**		**35-49**		**All age**		
**Variable**	**No. live births**	**Death n/N(%)**	**No. live births**	**Death n/N(%)**	**No. live births**	**Death n/N(%)**	**No. live births**	**Death n/N(%)**	**P-value**
**All *Birth order***	**3238**	**150/3238(4.63)**	**12294**	**346/12294(2.81)**	**2607**	**80/2607(3.07)**	**18139**	**576/18139(3.18)**	**<0.01**
First order	2549	102/2549(4.00)	7583	186/7583(2.45)	1430	37/1430(2.59)	11562	325/11562(2.81)	
Second order	689	48/689(6.97)	4711	160/4711(2.45)	1177	43/1177(3.65)	6577	251/6577(3.82)	
**Interval between first & second birth**	**689**	**48/689(6.97)**	**4711**	**160/4711(2.45)**	**1177**	**43/1177(3.65)**	**6577**	**251/6577(3.82)**	**<0.01**
Short birth interval (less than 33 months after the previous birth)	512	46/512(8.98)	2337	98/2337(4.19)	492	12/492(2.44)	3341	156/3341(4.67)	
Long birth interval (greater than 33 months after the previous birth)	177	2/177(1.13)	2374	62/2374(2.61)	685	31/685(4.53)	3236	95/3236(2.94)	

### Factors associated with the risk for neonatal death

Odds and adjusted odds ratios (aORs) for neonatal death are presented in Table 4. Findings from univariate analysis revealed association between neonatal death and numerous factors (maternal age at delivery, mother’s partner, sex of newborn, birth order and birth interval). These factors remained statistically significant even after entered in the multivariate model. As shown in Table 4, neonates of the teenage mothers (13–19 years) had 64% (aOR 1.64, 95% CI: 1.34-2.02) increased risk of dying during the neonatal period than those of older mothers (20–34 years). In addition, neonates in the second birth order had nearly 2 fold (aOR 1.85, 95% CI: 1.52-2.26) increased risk of dying than those in the first birth order. Furthermore, mothers with short birth intervals were 1.5 times (aOR = 1.5, 95% CI: 1.16-1.96) more likely to lose their child during the neonatal period than mothers who had long birth interval after the previous pregnancy. The risk of neonatal death was higher among male newborn than female counterpart (aOR = 1.34, 95% CI: 1.13- 1.58). We also found that presence of mother’s partner conferred protection against neonatal death, where mothers with partner present had 18% reduced risk of losing their child during neonatal period (aOR = 0.82 95% CI: 0.62-0.98).

**Table 4:** Logistic regression model for predictors of neonatal death in Ifakara HDSS: 2004–2009 (N = 18,139)

	**Univariate**			**Multivariate**		
**Factor**	**OR**	**95% CI**	**P-value**	**AOR**	**95% CI**	**P-value**
**Sex of newborn**						
Female (Ref.)	1.00			1.00		
Male	1.33	1.13-1.58	<0.01	1.34	1.13-1.58	<0.01
**Mothers’ age**						
Teenager mothers’ (20–34 years) (Ref.)	1.00			1.00		
Non-Teenage mothers’ (13–19 years)	1.68	1.38-2.04	<0.01	1.64	1.34-2.02	<0.01
Non-Teenage mothers’ (35–49 years)	1.09	0.85-1.40	0.48	1.1	0.86-1.41	0.463
**Birth order**						
First Births (Ref.)	1.00			1.00		
Second births	1.37	1.16-1.62	<0.01	1.85	1.52-2.26	<0.01
**Interval between first & second birth**						
Long birth interval (>33 months) (Ref)	1.00					
Short birth interval (<33 months)	1.62	1.25-2.10	<0.01	1.50	1.16-196	<0.01
**Maternal Education**						
Above primary education (Ref.)	1.00			1.00		
None or Primary education	1.24	0.98-1.56	0.072	1.2	0.94-1.53	0.136
**SES at birth**						
Poorest (Ref.)	1.00					
Poorer	0.92	0.71-1.20	0.557	-	-	-
Poor	0.94	0.73-1.22	0.644	-	-	-
Less Poor	0.97	0.75-1.25	0.795	-	-	-
Least Poor	1.09	0.84-1.42	0.516	-	-	-
**Place of delivery**						
Outside health facility (Ref.)	1.00					
Health facility	0.91	0.83-1.16	0.842	-	-	-
**Mother having partner**						
Absence (Ref.)	1.00			1.00		
Presence of mothers’ partner	0.8	0.65-0.98	0.031	0.82	0.66-0.98	0.049
**Season**						
Dry season (Ref.)	1.00					
Wet season	0.98	0.83-1.16	0.813	-	-	-

Ref. = Reference of the category, CI = Confidence Interval, OR = odds Ratio.

## Discussion

In Tanzania, the most recent Demographic and Health Survey conducted in 2010 indicated that neonatal mortality estimates declined from 32 deaths per 1000 in 2001–2005, to 26 deaths per 1000 in 2006–2010 [3,18]. This indicates 19% improvement in neonatal survival for the period from 2001 to 2010 [3]. Our findings indicates that the neonatal mortality rate was 32 per 1000 live birth during the period of 2004 to 2009 and declined from 36 per 1,000 live births in 2005 to 29 per 1,000 live births in 2009. The possible explanation for this decline could be attributed to different interventions related to maternal and newborns such as Integrated Management of Childhood Illness (IMCI) [19], and increased malaria control efforts, which lead to the decline of malaria morbidity in Kilombero and Ulanga [20].

The higher neonatal mortality rate among infants born to teenage mothers in our study corresponds with previous studies [21-23]. Usually adolescent mothers face financial and social problems that lead to less provision of child care [23]. Also, physiological immaturity of teenage mothers such as small uterus or narrow bony pelvis and lack of social experience on caring newborn can lead to more neonatal deaths [24]. Some scholars have argued that the neonatal deaths observed in teenage pregnancies might have been attributable to socio-demographic factors [25].

The association between birth order and increased neonatal death might be an artefact of overrepresentation of mothers with poor outcomes in their previous birth as reported by previous authors [26]. Previous studies also have shown that women who experience pregnancy loss or poor pregnancy outcome tends to go for next pregnancy after a short time to replace the previous pregnancy loss in order to achieve the desired family size, i.e. selective fertility or reproductive compensation [27-29]. This is an important challenge in reproductive epidemiology, as women who experienced poor outcomes are more likely to continue for the next pregnancy compared with those who had favourable birth outcomes. This results to overrepresentation of high risk group (women) in the subsequent pregnancies. Therefore, outcome of previous pregnancy is an important determinant for neonatal survival in the subsequent pregnancy including through shortening of the birth interval, it also influences the length of interval between pregnancies. The increased neonatal mortality among infants in the second birth order and among women with short birth interval could be explained by the effect of selective fertility.

Previous studies have showed that women who experienced preterm birth, delivery of low birth weight infants and neonatal death in their first pregnancy are more likely to have similar adverse outcome in subsequent pregnancies [26,30]. Since all these factors are associated with increased risk of neonatal death especially in young maternal age, the observed high risk of neonatal mortality in our study may in part be explained by recurrence of these factors in successive pregnancies [31].

In the present study, short birth interval was associated with increased risk of neonatal death compared with long birth interval [9,10]. It is generally accepted that if closely spaced births were delayed, particularly in countries where mortality and fertility are still high, child mortality levels would fall [29]. Birth intervals increase mortality of children in two ways: Children born after a short interval are likely to have mothers in poor health, and such children tend to have low birth weight and increased chances of neonatal mortality [30]. On the other hand, women with short intervals between two pregnancies have insufficient time to restore their nutritional reserves, a situation which is thought to adversely affect fetal growth and thereby increases risk of neonatal deaths.

Our study also showed that a mother with no partner at the time of birth had increased risk of neonatal death as compared to her counterpart who had a partner co-resident. Our findings are consistent with previous studies that assessed the impact of father’s involvement on child development, functioning and quality of life [32,33]. Evidence from the previous studies indicated that mothers with partner were more likely to provide their children with a healthy environment and nutritious food than mothers without partner, even when other conditions are similar [34,35].

We also found that neonatal mortality was higher for male newborns than females. Our results are in agreement with other studies that showed males had a higher odds of dying than females during the first month of life [36-39]. This increased hazard for newborn males may also be due to the large proportions of neonatal deaths occurring in the first week of life, which is the time when gender differences in neonatal mortality are most pronounced [36]. Biological factors that have been implicated with this increased risk of neonatal death in male infants include immunodeficiency [38] increasing the risks of infectious diseases in males, late maturity [36] resulting in a high prevalence of respiratory diseases in males, and congenital malformations of the urogenital system. Also higher mean birth weight in males as compared to females [39], which leads to more difficult births and more asphyxia and birth trauma, leading to higher neonatal mortality.

We found no evidence to suggest that delivery in health facilities is protective to the newborns. This observation concurs with a previous study in the same area [40] but is contrary to the expected neonatal survival gains conferred through institutional delivery [21]. In Tanzania, lack of safe delivery facilities, shortage of skilled providers as well basic equipment and supplies remain critical [41]. This situation is reflected in the observed high and slightly stable neonatal mortality rate despite some increase in the facility-based. Lack of adequate maternal and neonatal care at health facilities in time critical has been argued to be linked to deaths within the first day of life [42]. Findings from facility based studies in parts of north-eastern Tanzania that assessed unmet need for emergency obstetric care blamed poor quality of care for the negative maternal outcomes and high perinatal mortality [43]. Quality of delivery services and variations in newborn care practices were not included in these analyses but could affect the risk of neonatal deaths. A recent systematic review indicated that over three quartets of intrapartum-related deaths occurred in settings with weak health systems [44]. Scarcity of skilled providers, poor infrastructure and substandard quality of care are some of the critical components of such health systems that constrain progress in maternal and newborn survival [45].

### Strengths and limitations

This study utilized huge datasets from Health and Demographic Surveillance System which are continuously registered vital demographic events in a geographical defined area. Large sample size provided our study with a sufficient power to provide accurate statistical analysis across sub groups in the study population. On the other hand, findings from Health and Demographic Surveillance Systems data provides information to policy makers and program manager which can be translated into policy and practice.

This study has some limitations that need to be considered in interpreting the findings. First, self-reporting of neonatal deaths may result to under estimation of true neonatal mortality due to underreporting particularly for deaths that happened within the first day of life. Secondly, misclassification of stillbirth and early neonatal death, the demarcation between intrapartum stillbirth and early neonatal death is problematic, this leads to potential overestimation of early neonatal death as some stillbirths are regarded as early neonatal. Third, there are other possible factors associated with neonatal survival that were not available in the HDSS dataset, such as environmental, genetic factors, gestational age and birth weight.

## Conclusion

This study revealed that delayed maternal age at birth, presence of mother’s partner, birth order and birth interval are important factors which have an impact on neonatal mortality. These factors are amenable for strategy to promote and improve neonatal health and survival. Teenagers should access reproductive health information and services along with appropriate support to avoid early pregnancies. Women at high risk during first or second pregnancies need specialized care to reduce avoidable neonatal deaths. Sexual and reproductive health education programs should also promote use of family planning methods to enable women adhere to recommended birth interval and avoid unwanted pregnancies, increased age at marriage; and women empowerment in order to have control over their health. On the other hand, public health interventions directed at reducing neonatal death should address community, household and individual level factors such as birth interval, birth order and mother’s age that significantly influence neonatal mortality in Tanzania.

## Competing interests

The authors declare that they have no competing interests.

## Authors’ contributions

MS conceived the study idea, designed the study, carried out the statistical analyses, interpretation of the results, and drafted the manuscript. MA, SM, AS, DK, AM and RN participated in the design of the study, reviewed the manuscript. MJM provided technical support for interpreting results and reviewed it for intellectual content. MA was a coordinator of Ifakara Demographic Surveillance system during the study period. All authors read and approved the final manuscript.

## Pre-publication history

The pre-publication history for this paper can be accessed here:

http://www.biomedcentral.com/1471-2393/14/240/prepub

## References

[B1] UNICEF Levels and trends in child mortality report 2012 New York: United Nations Children’s Fund

[B2] UN Inter-agency Group for Child Mortality Estimation Levels and trends in child mortality 2013 1 30 http://www.childinfo.org/files/Child_Mortality_Report_2013.pdf

[B3] National Bureau of Statistics (NBS) and Macro International Inc Tanzania demographic and health Survey 2010 2011 Dar es Salaam, Tanzania: NBS and ICF Macro

[B4] Hinderraker SG Olsen BE Bergsjo PB Gasheka P Lie RT Kvale G Perinatal mortality in northern rural Tanzania Health Population Nutrition J 2003 21 8 17 12751669

[B5] Chen X Wen SW Fleming N Demissie K Rhoads GC Walker M Teenage pregnancy and adverse birth outcomes: a large population based retrospective cohort study 2007 Epidemiology: International Journal 10.1093/ije/dyl284 17213208

[B6] World Health Organization Issues in adolescent health and development 2004 Geneva: WHO

[B7] UNICEF Adolescence in Tanzania 2011 Dar es Salaam, Tanzania: United Nations Children’s Fund

[B8] Marchant T Schellenberg JA Nathan R Abdulla S Mukasa O Mshinda H Lengeler C Anaemia in pregnancy and infant mortality in Tanzania Trop Med Int Health 2004 9 2 262 266 10.1046/j.1365-3156.2003.01178.x 15040564

[B9] DaVanzo J Hale L Razzaque A Rahman M Effects of interpregnancy interval and outcome of the preceding pregnancy on pregnancy outcomes in Matlab, Bangladesh BJOG: An International Journal of Obstetrics and Gynaecology 2007 114 9 1079 1087 10.1111/j.1471-0528.2007.01338.x 17617195 PMC2366022

[B10] Stephansson O Dickman PW Cnattingius S The influence of interpregnancy interval on the subsequent risk of stillbirth and early neonatal death An International Journal of Obstetrics and Gynaecology 2003 102 1 101 108 10.1016/s0029-7844(03)00366-1 12850614

[B11] Ifakara DSS Tanzania 2011 access at http://www.indepthnetwork.org/dss_site_profiles/Ifakaraprofile.pdf on 24th May.2011

[B12] Schellenberg JA Mukasa O Abdulla S Marchant T Lengeler C Kikumbih N Mshinda HNR Sankon OA, Kahn K, Mwageni E, Ngom PNP Ifakara Demographic Surveillance System, Tanzania In population and Health in developing countries: Volume 1 population, Health and Survival at INDEPTH sites 2002 Ottawa, Canada: International Development Research centre 159 164

[B13] WHO Report of a WHO Technical Consultation on Birth Spacing 2007 Geneva, Switzerland: WHO

[B14] Ministry of Planning Economy and Empowerment National Population Policy 2006 Dar es Saaam, Tanzania: United Republic of Tanzania

[B15] Vyas S Kumaranayake L Constructing socio-economic status indices: how to use principal components analysis Health Policy and Planning 2006 21 459 468 10.1093/heapol/czl029 17030551

[B16] Bursac Z Gauss CH Williams DK Hosmer DW Purposeful Selection of Variables in logistic regression BioMedical Central Source Code for Biology and Medicine 2008 3 17 10.1186/1751-0473-3-17 PMC2633005 19087314

[B17] Corp S Stata: Release 11 2009 Statistical Software College station TX: Stata Corporation

[B18] National Bureau of Statistics (NBS) and Macro Internation Inc Tanzania Demographic and Health Survey 2004–05 2005 Dar es Salaam, Tanzania: NBS and ICF Macro

[B19] Sangber-Dery MD The role of birth order in infant mortality in Ifakara DSS area in rural Tanzania 2009 Johannesburg, South Afica: University of Witwatersrand

[B20] Alba S An Evaluation of Integrated Interventions to Improve Access to Malaria treatment in Tanzania (Access programme) 2010 University of Basel: PhD Thesis

[B21] Lawn JE Cousens S Zupan J 4 million neonatal deaths: when? Where? Why? Lancet 2005 365 9462 891 900 10.1016/S0140-6736(05)71048-5 15752534

[B22] Vandana S Joanne K Luke CM Subarna KK Steven CL Sharada RS Gary L Darmstadt JMT Young maternal age and the risk of neonatal mortality in Rural Nepal Arch Pediatr Adolesc Med 2008 162 828 835 10.1001/archpedi.162.9.828 18762599 PMC2535853

[B23] Markovitz BP Cook R Flick LHLT Socioeconomic factors and adolescent pregnancy outcomes: distinctions between neonatal and post-neonatal deaths? BioMedical Central J Public Health 2005 5 79 10.1186/1471-2458-5-79 PMC1190191 16042801

[B24] Wang CS Chou P Characteristics of males who father babies born to adolescents versus older adult women in Taiwan J Adolesc Health 2001 28 6 509 512 10.1016/S1054-139X(00)00180-4 11377995

[B25] Coall DA Chisholm JS Evolutionary perspectives on pregnancy: maternal age at menarche and infant birth weight Soc Sci Med 2003 57 1771 1781 10.1016/S0277-9536(03)00022-4 14499504

[B26] Hamisu M Salihu HM Salinas A August EM Mogos MF Weldeselasse H Whiteman VE Small Size for Gestational Age and the Risk for Infant Mortality in the Subsequent Pregnancy Ann Epidemiol 2012 22 11 764 771 10.1016/j.annepidem.2012.07.003 22858049 PMC3541006

[B27] Quamrul HC Rafiqul I Karmal H Effects of Demographic Characteristics on Neonatal, Post neonatal, Infant and Child Mortality Curr Res J Biol Sci 2010 2 2 132 138

[B28] Rahman MM Abidin S Factors Affecting Neonatal Mortality in Bangladesh J Health Manag 2010 12 2 137 152 10.1177/097206341001200203

[B29] Conde-Agudelo A Rosas-Bermudez A Kafury-Goeta AC Effects of birth spacing on maternal health: A systematic review Am J Obstet Gynecol 2007 196 4 297 308 10.1016/j.ajog.2006.05.055 17403398

[B30] Rutstein S Effects of preceding birth intervals on neonatal, infant, and under-five years mortality and nutritional status in developing countries: evidence from the demographic and health surveys Int J Gynaecol Obstet 2005 89 suppl 1 S7 S24 15820369 10.1016/j.ijgo.2004.11.012

[B31] Mahande MJ Daltveit AK Obure J Mmbaga BT Masenga G Manongi R Lie RT Recurrence of preterm birth and perinatal mortality in northern Tanzania: registry-based cohort study Trop Med Int Health 2013 doi:10.1111/tmi.121111 10.1111/tmi.12111 PMC3749445 23581495

[B32] Horn WF Sylvester T Father Facts 2002 4 National Fatherhood Initiative http://ncfy.acf.hhs.gov/library/2002/father-facts-fourth-edition

[B33] Sartorius B Kahn K Collinson MA Vounatsou PTS Survived infancy but still vulnerable: spatial-temporal trends and risk factors for child mortality in the Agincourt rural sub-district, South Africa, 1992–2007 Geospat Health 2011 5 2 285 295 21590679 10.4081/gh.2011.181 PMC3523210

[B34] Hani KA Parents’ death and its implication for child survival Rev Brasileira de Crescimento Desenvolvimento Humano 2011 21 3 769 770 PMC4501914 26185355

[B35] Alonso V Fuster VLF Causes of neonatal mortality in Spain (1975–98): influence of sex, rural–urban residence and age at death J Biosoc Sci 2006 38 4 537 551 10.1017/S0021932005026957 16762089

[B36] Shakya K McMurray C Neonatal mortality and maternal health care in Nepal: searching for patterns of association J Biosoc Sci 2001 33 1 187 105 10.1017/s0021932001000876 11316397

[B37] Eshima N Tokumaru O Hara S Bacal KKS Age-Specific Sex-Related Differences in Infections: A Statistical Analysis of National Surveillance Data in Japan PLoS One 2012 7 7 e42261 10.1371/journal.pone.0042261 22848753 PMC3407080

[B38] Adolfo MR Maria JG de Marillia Carvalho L Silvia W Savinho SBC Risk factors for neonatal mortality among children with low birth weight Rev Saude Publica 2009 43 2 1 9 10.1590/s0034-89102009005000004 19225697

[B39] Mmbaga BT Lie RT Olomi R Mahande MJ Kvåle G Daltveit AK Cause-specific neonatal mortality in a neonatal care unit in Northern Tanzania: a registry based cohort study BioMedical Central Pediatrics; 2012 12 1 116 10.1186/1471-244X-12-116 PMC3469393 22871208

[B40] Nathan R Mwanyangala MA Survival of neonates in rural southern Tanzania: does place of delivery or continuum of care matter? BioMedical Central Pregnancy and childbirth 2012 12 18 10.1186/1471-2393-12-18 PMC3384458 22439592

[B41] Schellenberg JAR Mrisho M Manzi F Shirima K Mbuya C Mushi AK Katende SC Alonso PL Mshinda H Tanner M Schellenberg D Health and survival of young children in southern Tanzania BioMedical Central Public Health 2008 8 194 1 17 10.1186/1471-2458-8-194 PMC2442074 18522737

[B42] Lawn J Kerber K Enweronu-laryea C Batwman M Newborn survival in low resource settings: are we delivering? BJOG: An International Journal of Obstetrics and Gynaecology 2009 116 suppl 1 S49 S59 10.1111/j.1471-0528.2009.02328.x 19740173

[B43] Olsen OE Ndeki S Norheim OF Availability, distribution and use of emergency obstetric care in northern Tanzania Health Policy Plan 2005 20 3 167 175 10.1093/heapol/czi022 15840632

[B44] Lawn JE Kinney M Lee AC Chopra M Donnay F Paul VK Bhutta ZA Bateman M Darmstadt GL Reducing intrapartum-related deaths and disability: can the health system deliver? International Journal of Obstetrics and Gynaecology 2009 107 Suppl 1 S123-140 S140-122 10.1016/j.ijgo.2009.07.021 19815205

[B45] Koblinsky M Matthews Z Hussein J Mavalankar D Mridha MK Anwar I Achadi E Adjei S Padmanabhan P Marchal B De Brouwere V vanLerberghe W Going to scale with professional skilled care Lancet 2006 368 9544 1377 1386 10.1016/S0140-6736(06)69382-3 17046470

